# Editorial: Single-domain antibodies—biology, engineering and emerging applications, volume II

**DOI:** 10.3389/fimmu.2024.1537054

**Published:** 2024-12-12

**Authors:** Kevin A. Henry, Greg Hussack, Jan Gettemans, Cory L. Brooks

**Affiliations:** ^1^ Human Health Therapeutics Research Centre, National Research Council Canada, Ottawa, ON, Canada; ^2^ Department of Biochemistry, Microbiology and Immunology, University of Ottawa, Ottawa, ON, Canada; ^3^ Department of Biochemistry, Faculty of Medicine and Health Sciences, Ghent University, Ghent, Belgium; ^4^ Department of Chemistry and Biochemistry, California State University Fresno, Fresno, CA, United States

**Keywords:** single-domain antibody, nanobody, VHH, VNAR, antibody engineering, antibody therapy

Since the first volume of this Research Topic was published in 2017 (1), the single-domain antibody (sdAb) field has evolved dramatically. The first sdAb-based drug, the anti-von Willebrand factor caplacizumab (Cablivi), was approved for the treatment of acquired thrombotic thrombocytopenic purpura by the EMA and FDA in 2018 and 2019, respectively (2). The emergence of SARS-CoV-2 and response to the resulting COVID-19 pandemic firmly established the antiviral neutralization potency of sdAbs, especially well-designed multiparatopic molecules (3). The fields of cell therapy and chimeric antigen receptor (CAR) design have exploded, leading to seven FDA-approved CAR-T cell therapies including ciltacabtagene autoleucel (Carvykti), a BCMA-targeted tandem sdAb-based product for the treatment of relapsed or refractory multiple myeloma (4). Recent approvals in Japan of ozoralizumab (Nanozora) (5), a trimeric sdAb targeting TNF and serum albumin for the treatment of rheumatoid arthritis, and in China of envafolimab (6), a PD-L1-specific sdAb fused to IgG1 Fc for various advanced solid tumours, highlight a growing momentum. Clearly, sdAbs are no mere biological curiosities or niche research objects but an entirely distinct class of binding molecules that are now coming into their own.

Some of the themes of the first volume also extend to the second. The advantages of sdAbs over conventional antibodies and their fragments in a variety of applications are clearly illustrated in the 12 original research articles and 2 reviews of this Research Topic, which together provide a snapshot of trends and recent developments. In particular, many of the articles in the second volume investigated uses of sdAbs for non-invasive imaging and as diagnostics, often to detect SARS-CoV-2.

## Biology of single-domain antibodies

One original research article addressed the fundamental properties of sdAbs. In the largest study of this type conducted to date, Gordon et al. compared the structures of 345 sdAb:antigen complexes and 892 conventional antibody:antigen complexes with the goal of understanding the potentially distinct mechanisms of antigen recognition by sdAbs. In agreement with prior studies, the results of this analysis show that the paratopes of sdAbs are smaller than those of conventional antibodies; however, neither differences in paratope amino acid composition nor differences in the size (defined as the number of residues), amino acid composition or accessibility of epitopes targeted by sdAbs were evident. The explanation for this apparent contradiction is that within smaller sdAb paratopes, a longer complementarity-determining region 3 (CDR3) loop contributes a greater number of interactions per residue and framework residues are more likely to play a role in binding.

## Discovery and engineering of single-domain antibodies

One original research article investigated a new approach for camelid sdAb discovery. While many groups have integrated high-throughput sequencing of antibody repertoires into existing discovery pipelines in which antigen reactivity of individual clones is evaluated *in vitro*, Matsuda et al. developed a predictive algorithm to identify antigen-specific sdAbs without *in vitro* screening by longitudinal sequencing and phylogenetic analysis of the peripheral sdAb repertoire. The basis for identifying antigen-specific sdAbs is the accumulation of somatic hypermutations and high turnover rates within clonal families during the process of affinity maturation. While preliminary characterization of antigen-specific sdAbs recovered using this strategy showed variable binding data across assays, and concurrent immune responses mounted against non-immunizing antigens including pathogens would be expected to confound predictions, the encouraging overall results indicate it may one day be possible to accurately identify antigen-specific sdAbs following immunization via sequencing of the peripheral blood repertoire.

Two original research articles examined the ability of sdAbs, or even smaller antibody-derived fragments, to extend the serum persistence of biologics via binding to serum albumin. Harmsen et al. isolated and characterized sdAbs from the repertoire of a llama immunized with dog and horse serum albumin. Unlike previous efforts in this regard, the sdAbs bound the albumins of various animal species including horse, dog, cat and swine – but did not recognize those of human or mouse – and extended the half-life of a tetanus toxin-specific sdAb in pigs and horses. These sdAbs would be useful for therapeutic studies of molecules with intrinsic short half-lives in these animals. Adams et al. identified bovine ultralong CDRH3s (‘knob domains’) that mediate autonomous high-affinity binding to human or mouse serum albumin in the absence of the remainder of the parental bovine antibody. These albumin-specific knob domains could be introduced recombinantly into the VH framework region 3 D-E loop (also known as the ‘CDR4 loop’) of a TNF-specific Fab or chemically via conjugation to an IL-17 inhibitory peptide resulting in dual antigen recognition. In the former case, half-life extension of the bispecific anti-TNF Fab bearing the anti-mouse serum albumin knob domain was observed in mice. These results reinforce the utility of bovine knob domains as a unique class of antigen recognition units and demonstrate that serum albumin recognition and half-life extension can be conferred by incorporation of a 4-5 kDa antibody-derived polypeptide.

## Single-domain antibodies for non-invasive imaging

Four original research articles focus on applications of sdAbs as non-invasive imaging tracers, taking advantage of their high affinity binding and rapid clearance from circulation. Benloucif et al. generated llama anti-MSLN sdAbs that do not compete with MUC16 or amatuximab for MSLN binding and evaluated their ability to detect MSLN expression using fluorescence (ATTO 647N labelling) or PET/CT (^68^Ga labelling). The resulting tracers showed preferential uptake in tumors expressing high levels of MSLN and are compatible with monitoring of available therapies. Zeven et al. discovered novel llama anti-TIGIT sdAbs and designed an scFv based on vibostolimab, labeled these molecules with ^99m^Tc, and evaluated their ability to detect TIGIT expression using SPECT/CT imaging. Despite stronger binding to TIGIT-expressing PBMCs by the scFv, the sdAbs showed superior *in vivo* tumor labelling, potentially due to their enhanced stability and/or tissue penetration. Wagner et al. describe novel alpaca sdAbs against SIRPα, some of which block the CD47-SIRPα interaction. One of the non-blocking sdAbs was ^64^Cu labeled and used to visualize tumor infiltration by myeloid cells by PET/MR. Theranostic applications of these sdAbs can be envisioned by modifying the radioisotope used.

Most *in vivo* imaging tracers incorporate a single label that is either fluorescent or radioactive. Declerck et al. produced bimodal anti-uPAR sdAb tracers by conjugating ^99m^Tc and IRDye800CW site-specifically to C-terminal His_6_ and Cys tags, respectively. The combination of fluorescence and SPECT/CT imaging may help overcome the limitations of each approach (*e.g.*, limited tissue penetration of fluorescent signals, imprecision of gamma probing for intra-operative decision making).

## Single-domain antibodies against SARS-CoV-2 and other pathogens

Two reviews, two original research articles and one brief research report explore applications of sdAbs for diagnosis and treatment of infections, primarily SARS-CoV-2. Cabanillas-Bernal et al. comprehensively review recent studies using shark VNARs as antiviral agents against SARS-CoV-2. Meanwhile, De Greve and Fioravanti extensively review the broader literature on camelid sdAbs for treatment of microbial infections by bacteria and viruses, an expansive and constantly evolving topic.

One original research article and one brief research report describe sdAb-based diagnostic assays for SARS-CoV-2. Segovia-de los Santos et al. developed a diagnostic luciferase assay using all recombinant reagents in which streptavidin-coated plates are loaded with a biotinylated nucleocapsid-specific sdAb, antigen is captured, and bound antigen is detected using a second non-competitive sdAb fused to NanoLuc. The assay was validated using 144 clinical samples from 2022 when Omicron (B.1.1.529) was the dominant variant in Uruguay. Goldman et al. developed a Luminex MagPlex assay in which SpyCatcher-coated magnetic beads are loaded with SpyTagged nucleocapsid-specific sdAb, antigen is captured, and bound antigen is detected using a second non-competitive reporter sdAb that is biotinylated. In both studies the oriented (rather than randomly adsorbed) sdAb matrices are key to increased sensitivity of the assays.

One original research article showcases the therapeutic potential of engineered sdAbs to bind and neutralize emerging SARS-CoV-2 variants. Following immunization of transgenic mice producing heavy chain-only antibodies, Du et al. constructed a hexavalent antibody consisting of two tandemly arrayed copies of an RBD-specific sdAb and one NTD-specific sdAb fused N- and C-terminally, respectively, to human IgG1 Fc. The enhanced avidity of this molecule permitted neutralization of Omicron sublineages that escaped neutralization by the individual component sdAbs as bivalent sdAb-Fc fusions.

## Conformation-specific single-domain antibodies

One original research article tackled the challenging problem of developing antibodies that are able to specifically recognize particular conformational states of proteins. Zupancic et al. identified llama sdAbs from yeast-displayed libraries using MACS- and FACS-based selection that preferentially recognize aggregated (fibrillar) tau over soluble monomeric tau. These sdAbs were able to recognize tau aggregates in brain samples from transgenic mice as well as from patients with tauopathies, and may have diagnostic or therapeutic applications in neurodegenerative diseases.

## Final thoughts

Regulatory approval of four sdAb-based drugs (three biologics and one CAR-T cell) has substantially altered perceptions and attitudes towards these molecules in the medical and scientific communities. With mainstream acceptance has come increased visibility and interest. However, efforts and investment continue to center on discovery and biotechnological applications of sdAbs, and much work still remains to understand the basic immunobiology of these unique molecules as well as how to generate, engineer, characterize and manufacture them most effectively.

The editors would again like to thank all contributors for the many excellent submissions to this Research Topic, as well as the reviewers and the *Frontiers in Immunology* editorial office.
